# Combined computational and experimental studies on cysteine-sulfadiazine adduct formation

**DOI:** 10.3906/kim-1908-62

**Published:** 2020-04-01

**Authors:** Nursel ACAR SELÇUKİ, Emine COŞKUN, Ender BİÇER

**Affiliations:** 1 Department of Chemistry, Faculty of Science, Ege University, Bornova, İzmir Turkey; 2 Department of Chemistry, Faculty of Arts and Science, Ondokuz Mayıs University, Atakum, Samsun Turkey

**Keywords:** Cysteine-sulfadiazine adduct, nucleophilic attack, density functional theory

## Abstract

The electrochemical characterization of sulfadiazine-cysteine (SD-CYS) adduct formation was performed in phosphate buffer (pH 7) on the basis of voltammetric current and peak potential measurements. Due to the association of cysteine with sulfadiazine, the reduction peak currents of mercuric and mercurous cysteine thiolates decreased and their peak potentials simultaneously shifted to less negative potentials. By using the current changes of mercurous cysteine thiolate, it was determined that cysteine and sulfadiazine are associated with a 1:1 stoichiometry with a conditional association constant of 1.99 ×104 M−1 . In addition to experimental studies, a computational approach was carried out to study the geometrical parameters, electron densities, and UV-Vis absorption spectra of sulfadiazine and SDCYS adduct in water. Calculated (B3LYP/6-311++G(d,p) level) and experimental UV-Vis absorption spectra of the compounds were found to be in good agreement in water. The computational study suggests that cysteine bound to the C(5) on the pyrimidine ring via SH-group nucleophilic attack. Computational results reveal that sulfadiazine and its derivatives effectively bind cysteine and may lead to new molecules/drugs to target cysteine.

## 1. Introduction

Sulfadiazine (SD) is used for curing infections caused by gram-positive and gram-negative organisms [1] and it belongs to the sulfonamide category [2]. The sulfonamides are found in blood in three different forms: proteinbound, conjugated (acetylated and possibly others), and free [3]. The drug acts by the diffusion of its unbound form through the circulatory system and interacts with action sites [2].

Biological processes inside the human body are directly affected by drug–protein interactions [4]. Drug– protein interactions are usually investigated by using small molecular systems in which amino acids, peptides, and their derivatives are used to mimic proteins in aqueous solutions [4–7]. These simpler systems are more useful as they simplify the investigation of interactions in aqueous solutions by decreasing the number of functional groups in proteins [4]. Sulfadiazine and other sulfonamides are inhibitors of the enzyme dihydropteroate synthase (DHPS) [8,9].

There is great interest in biomedical research to take advantage of the various structural interactions between amino acids and antibiotics. However, some side reactions may cause problems. For example, when the substituent groups of drugs interact with amino acids, the drugs will not work properly, or drug–amino acid complexes may display different effects rather than the expected drug properties. Therefore, knowledge of the interactions between drugs and amino acids will give rise to ideas about drug design. Although there are other amino acids chosen as drug targets, the presence of a thiol group makes cysteine a primary research interest [10,11].

Cysteine (CYS) is one of the two sulfur-containing proteinogenic amino acids and is involved in some important cellular functions like detoxification, protein synthesis, and metabolism [12,13]. The sulfhydryl (-SH) group of CYS is essential for proteins’ and enzymes’ biological functions and it exists as thiol (-SH) or thiolate (-S−) forms at neutral pH [12,13]. The acidity (Ka) of the thiol group regulates the equilibrium and hence the relative amount of S− with respect to SH. Accordingly, pKa for the sulfhydryl group of CYS is 8.30 [14]. At pH 7.0, both thiol and thiolate groups coexist in the medium; however, CYS probably reacts in its deprotonated form. The free energy cost for deprotonation depends on the pKa and pH values [15–17]. Thus, for sulfadiazine-cysteine (SD-CYS) adduct formation, the pH of the medium was selected as 7.0. Also, CYS has been identified as a valuable biomarker [18]. There are therefore numerous research studies focused on the interactions of CYS with folates, catechol, quinoids, benzoquinones, and some drugs [18–29].

Although there are many studies on the interactions of SD with some compounds (cyclodextrins, glycine, leucine, aspartic acid, glutamic acid, arginine, human serum albumin, peptide amides, lysozyme, DNA, watersoluble proteins) [1–3,30–35], adduct formation between SD and CYS has not been addressed in the literature. Electrochemical techniques are frequently used to study the effects of electroactive species upon molecular interactions [36–38]. In the present study, the binding of SD to CYS was investigated in a neutral aqueous medium by means of square-wave voltammetry, UV-Vis spectroscopy, and computational studies together with optimized geometries. The current study focuses on covalent bonding between SD and CYS, which will provide useful information for the development of new molecules or drugs targeting CYS.

## 2. Materials and methods

### 2.1. Reagents and equipment

Square-wave voltammograms (SWVs) were recorded using an EG&G PAR 384B Polarographic Analyzer combined with the EG&G PARC 303A SMDE. The electrode system used consisted of a hanging mercury drop electrode (working electrode), Ag/AgCl/KClsat. (reference electrode), and Pt wire (auxiliary electrode). ECDSOFT software was used to obtain voltammograms on a laptop computer [39]. A Janway 3010 pH meter was used for all pH measurements. UV-Vis absorption spectra were obtained from a PerkinElmer Lambda 35 spectrophotometer. FTIR-ATR spectra were obtained by a PerkinElmer Spectrum 100 FT-IR Spectrometer.

L-CYS was purchased from Merck and SD was purchased from Sigma. All other reagents were of analytical reagent grade. SD was dissolved in methanol. Stock solutions of other reagents were prepared daily by dissolving their appropriate amounts in ultrapure water (specific resistivity: 18.2 MΩ cm). Phosphate buffer was also prepared in ultrapure water and its pH (pH 7.0) was adjusted by addition of 0.5 M NaOH solution.

### 2.2. Synthesis of SD-CYS complex

**Figure 1:**
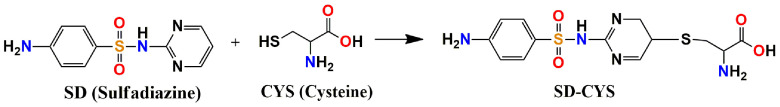
Molecular structures and reaction scheme of the investigated compounds.

A mixture of 0.0001 mol SD and 0.0001 mol L-CYS was dissolved in 30 mL of methanol (70%). This solution was continuously stirred with a constant temperature about 40 °C for 4 h. After the evaporation of most of the solvent at room temperature for 4–5 weeks, a white solid compound (SD-CYS adduct) was obtained and dried at room temperature. The simplified reaction is given in Figure 1.

### 2.3. Electrochemical procedure

Phosphate buffer (10 mL, 0.02 M, pH 7.0) was added to the electrochemical cell and degassed with N2 for 300 s. The voltammogram was recorded by applying the potential scan toward the positive direction. After the background voltammogram was obtained, CYS was added and the voltammogram was obtained by the same procedure. Appropriate amounts of SD were then added to the electrochemical cell and the changes were followed in voltammograms. All electrochemical experiments were carried out at room temperature.

### 2.4. Computational details

Spartan08 [40] was used to obtain initial structures by conformational analysis. The geometry optimizations were performed with Gaussian09 [41] using density functional theory (DFT) [42–44] with the ωB97XD functional [45] in combination with the 6-311++G(d,p) basis set. This functional was chosen as it has long-range terms and can calculate weak dispersion interactions [45]. Gaussview5.0 [46] was used for visualization. The minimum nature of all optimized structures was verified with frequency calculations at the same level. Time-dependent DFT (TD-DFT) calculations were performed to calculate the UV-Vis absorption spectra (N = 100 states) and molecular orbital energies (EHOMO , ELUMO , ΔEH−L) using the ground-state optimized geometries. All TDDFT calculations were performed with Becke’s 3-parameter exchange and Lee–Yang–Parr correlation functionals (B3LYP) [47] in combination with the 6-311++G(d,p) basis set. TD-DFT computations were repeated with the ωB97XD functional with the same basis set to obtain UV-Vis spectra and both computational results were compared with experimental UV-Vis absorption spectra. To mimic the real systems, all calculations were done in solution. The polarizable continuum model (PCM) [48] was used in all DFT and TD-DFT calculations to investigate solvent effects on the electronic transitions in solution (water).

## 3. Results and discussion

### 3.1. Voltammetry measurements

**Figure 2:**
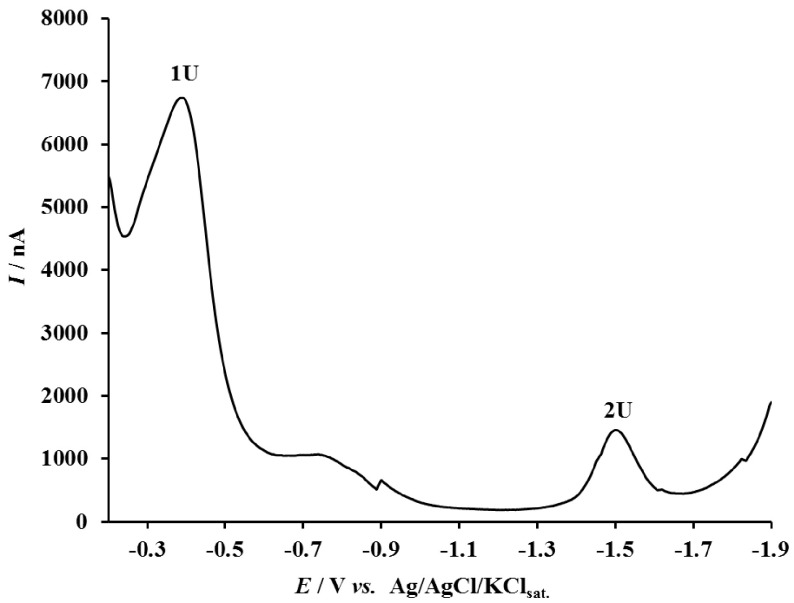
SWV of 1.0 ×10−4 M SD in phosphate buffer solution of pH 7.0 (other experimental conditions: equilibrium time of 5 s, scan increment of 4 mV, and frequency of 120 Hz).

The nucleophilic substitution reaction of CYS on SD was studied by square-wave voltammetry. Figure 2 shows the square-wave voltammogram of 1.0 ×10−4 M SD in phosphate buffer solution of pH 7. As can be seen in Figure 2, SD shows two cathodic peaks at –0.396 (1U) and –1.500 V (2U), corresponding to the reduction of Hg(II)-sulfadiazine adsorbed on the mercury electrode [49,50] and the reduction at the Ar-SO2 NH- group in a single irreversible reduction step [51–53], respectively.

**Figure 3:**
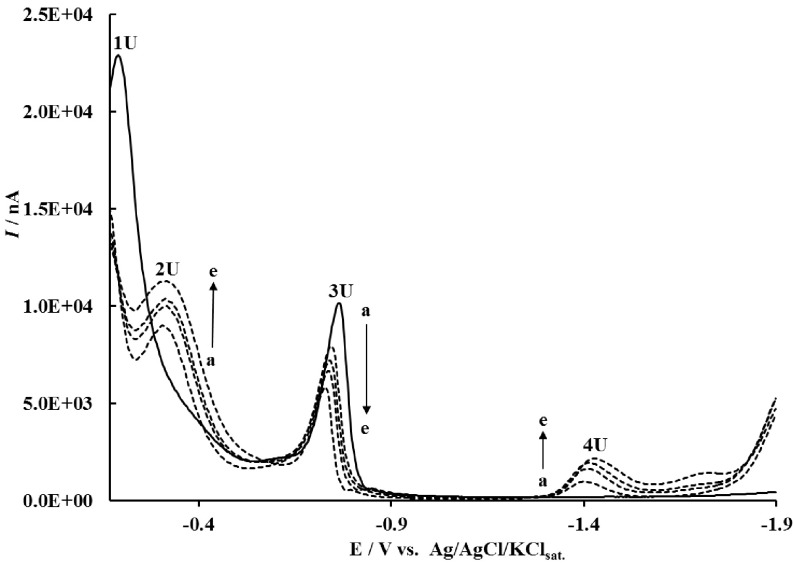
SWVs of 1.0 ×10−5 M CYS in the presence of a) 0, b) 1.0 ×10−4 , c) 2.8 ×10−4 , d) 3.6 ×10−4 , and e) 6.0 ×10−4 M SD in phosphate buffer solution of pH 7.0 (other experimental conditions: equilibrium time of 5 s, scan increment of 4 mV, and frequency of 120 Hz).

On the other hand, square-wave voltammograms obtained from 1.0 ×10−5 M CYS in the absence and presence of SD are shown in Figure 3. In the phosphate buffer solution of pH 7, CYS gave two well-developed cathodic peaks in the absence of SD (Figure 3). These peaks at –0.190 and –0.766 V (Figure 3) can be explained by the reductions of mercuric (1U) and mercurous cysteine thiolates (3U), respectively [54,55].

Upon addition of SD, the reduction potentials of the mercuric and mercurous thiolates shifted positively and their cathodic peak currents started to decrease (Figure 3), which suggested the nucleophilic attack of CYS to SD, or in other words the formation of the SD-CYS adduct. At the same time, upon addition of SD to CYS solution, newly appeared cathodic peaks at –0.342 (2U) and –1.426 V (4U) were increased gradually (Figure 3). New cathodic peaks (2U and 4U in Figure 3) correspond to the reductions of mercury salt and the electroactive Ar-SO2 NH- group of the SD-CYS adduct at less negative potentials than those of free SD. This behavior is in agreement with that reported by Proková and Heyrovský for thiols and their folate adducts [19].

**Figure 4:**
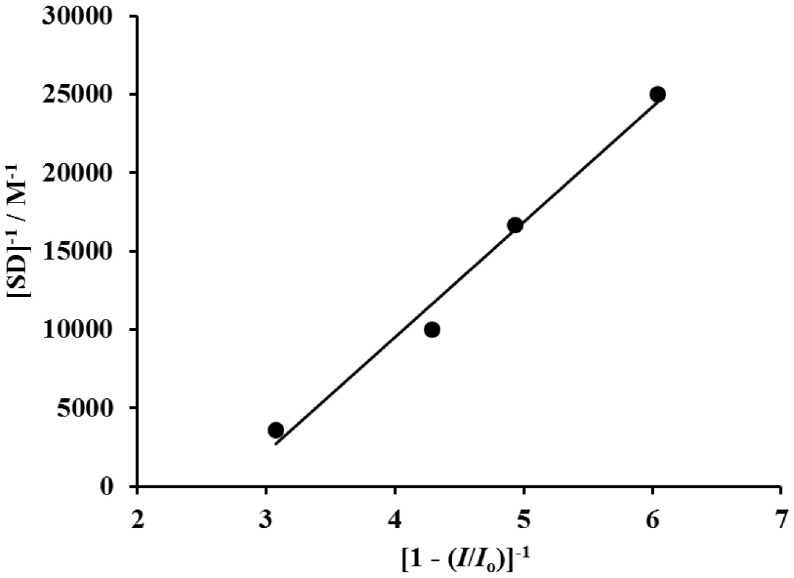
Plot of [SD]−1 vs. [1 – (I /Io) ]−1 for SD-CYS adduct.

According to the decrease in the peak current of mercurous cysteine thiolate with increasing concentrations of SD (Figure 3), the binding constant was calculated according to the following equation [56]:

[SD]-1=K(1-A)[1-(I/I0)]-1-K

where K is the binding constant, Io and I are the peak currents in the absence and presence of SD, and A is the proportionality constant. The plot of [SD]−1 versus [1 – (I /Io) ]−1 was drawn (Figure 4) and the value of K is calculated as 1.99 ×104 M−1 (R2 = 0.9855) using the intercept from this graph. The calculated association constant of 1.99 ×104 M−1 is attributed to a reversible inhibition [57] and a moderate-strength interaction [58]. The irreversible inhibition process is controlled by the barrier height: for a sufficiently high barrier the crossing is slower than the duration of the experiment. If the whole enzyme is taken into account, use of the simplified EVB model is particularly effective in cases with high barriers and many protonation sites in a computational approach [59].

It is well known that a SH-group may be added to the pyrimidine C(5) = C(6) bond by the CYS nucleophilic attack on the substrate [60]. Also, the interaction of thiyl radicals with the C5-C6 double bond in pyrimidines was reported by Wójcik et al. [61]. Moreover, it was observed that at the formation of uracil-CYS heterodimer, the amino acid was added to the 5 position rather than the 6 position of uracil with the formation of 5-S-cysteine-6-hydrouracil [62]. In this study, we also suggest that the SD-CYS adduct comes from the nucleophilic attack of the SH group of CYS to the C(5) = C(6) bond of pyrimidine at the SD molecule.

### 3.2. ATR-FTIR study

**Figure 5:**
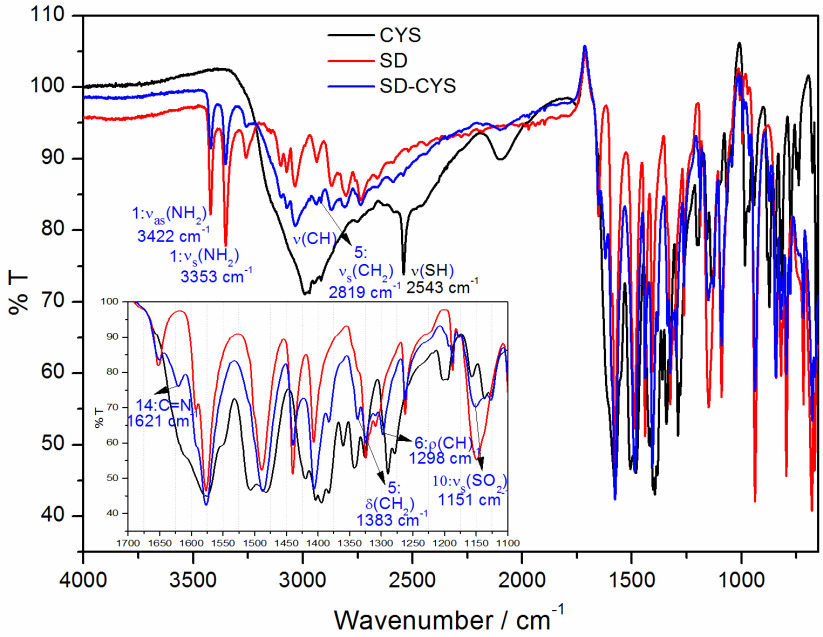
Experimental FTIR spectra of the investigated compounds (frequencies between 1100 and 1700 cm−1 are shown separately and important frequencies for SD-CYS complex are written).

**Figure 6:**
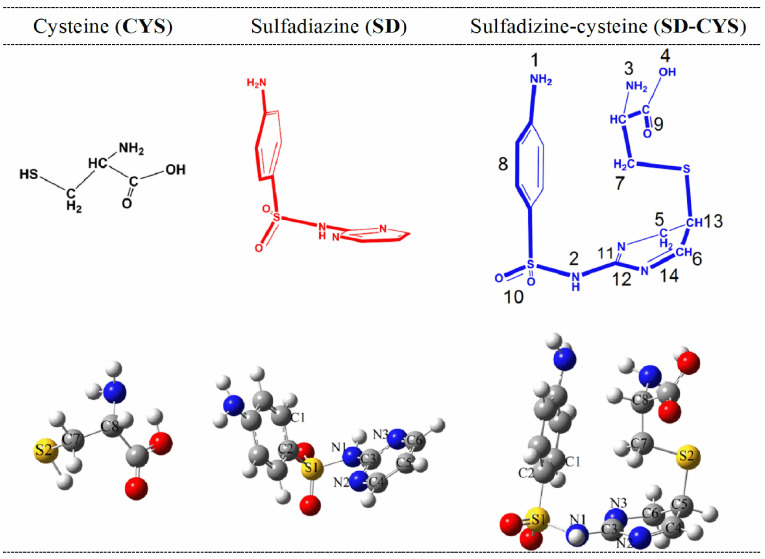
Optimized geometries of the investigated molecules in water at ωB97XD/6- 311++G(d,p) level.

The infrared spectra of SD, CYS, and SD-CYS adducts are shown in Figure 5. Figure 6 displays the optimized geometries of the reactants and the product; selected important bonds and atoms are numbered for simplification. The characteristic bands of SD (Figure 5) are seen at 3422 and 3353 cm−1 for symmetric stretching and asymmetric stretching of NH2 (vs (NH2) and vas (NH2)) . In the 2750–3150 cm−1 region of the spectrum, there are C-H stretching bands (Figure 5). A new peak in the same region appeared at 2819 cm−1 (Figure 5, bond 5) for symmetrical vibration of CH2 (vs (CH2)) due to pyrimidine deformation. The bands at 1575, 1490, 1440, and 1410 cm−1 are ring skeletal vibrations. The bands at 1325 and 1150 cm−1 belong to the -SO2 -Ngroup. The bands at 1585 and 1621 cm−1 are assigned to vC=N [1,63]. The new peaks are observed at 1383 and 1298 cm−1 . The peak observed at 2543 cm−1 in the ATR-FTIR spectrum of CYS (Figure 5) is due to the SH stretching [64–66]. Since this peak is not observed in the ATR-FTIR spectrum of the SD-CYS adduct (Figure 5), this observation may lead to the conclusion that the thiol hydrogen atom moved to the C5-C6 double bond on SD. Moreover, some important differences were observed in the ATR-FTIR spectrum of the SD-CYS adduct. In the range of 3500–2750 cm−1 , although the bands are similar, mainly decreases in intensity and small variations in position were obtained.

### 3.3. Computational results

Free CYS represents only truncated protein. However, by considering the entire enzyme, properties, and especially kinetics, would be changed (the rate constant will probably increase relative to the corresponding reaction in aqueous solution). Multiscale ab initio QM/MM is typically computationally too demanding and does not allow for well-converged reaction profiles. Empirical valence bond (EVB) is a method developed for calculating free energies of activation for enzyme reactions and reactions in solution [67]. In the current study, a simple mechanism for the reaction of free CYS with SD is investigated and the free energy values are calculated by DFT and PCM methods as explained in Section 2.

 There are two possible sites for the complex formation reaction between SD and CYS. The first is between the SH group of CYS and the pyrimidine of SD (S-bridged structure previously explained), and the second is between the carbonyl group of CYS and the phenyl-NH2 group of SD. Approximately, 100 conformers for both possibilities were optimized in water. Table S1 in the Supplemental Information displays E+ZPE energies and the optimized geometry of the most stable NH2 -bridged SD-CYS complex in water. The results for the SHbridged SD-CYS complex are given in Table S2 in gas phase and in water. Computational results revealed that the SH binding site forms the most stable complex, in agreement with experimental results. The NH2 -bridged complex forms in a condensation reaction producing 1 mol of water as a second product. Therefore, summed energies of the NH2 -bridged complex and water are compared with the energy of the S-bridged complex. As seen from Tables S1 and S2, the S-bridged complex is more stable than the NH2 -bridged complex and this confirms the experimentally observed structure. The optimized geometries of the most stable structures for the reactants and product are shown in Figure 6.

Table 1 lists the total energy and free energy differences of the investigated molecules for the reaction given in Figure 1. Table S3 shows dipole moments (μ, in debyes), sum of total electronic energies and zero point energies (E+ZPE), and selected dihedral angles of SD and SD-CYS for ground-state geometries optimized at the ωB97XD/6-311++G(d,p) level of theory in water. Dipole moment of the complex increased significantly with the inclusion of NH2 and OH groups from CYS. Bond distances changed slightly in the complex compared to the initial monomers. The S2-C7-C8 angle (114.12°) decreased by 4.37°in the complex compared to CYS. On the other hand, dihedral angles show significant differences between SD and SD-CYS molecules. Another important change is the distortion of the planarity for the pyrimidine ring in SD because of the newly formed S-C bond. The first step of the reaction is the formation of INT and it has a free energy barrier of 22 kcal/mol (Table 1; Figure S1). The transition state (TS1) is a late transition state and is isoenergetic with the INT. These energy values indicate that this step is reversible. The second step is the formation of the product (SD-CYS adduct) with the addition of H cation to the pyrimidine ring. The transition state (TS2) for this step could not be obtained even though all available options in Gaussian09 were used. This step is highly exergonic and the product is more stable than the INT by 190 kcal/mol. The second step is irreversible and once the product is stable the reaction terminates.

**Table 1:** 

	E+ZPE (Hartree)	E+ΔG (Hartree)	^a^ΔE (kcal/mol)	^b^ΔΔG (kcal/mol)	Distances (Å) (C…..S)
Reactants (SD+CYS)	–1875.84072	–1875.91625	0.00	0.00	
TS1	-1875.82947	-1875.88075	7.06	22.28	2.038
INT	-1875.82780	-1875.87993	8.10	22.79	1.978
product	-1876.29516	-1876.34765	-190.01	-190.23	1.872

^a^: ΔE = [E+ZPE(SD-CYS) – E+ZPE(SD) – E+ZPE(CYS)]. ^b^: ΔΔG = [E+ΔG(SD-CYS) – E+ΔG(SD) – E+ΔG(CYS)].

**Figure 7:**
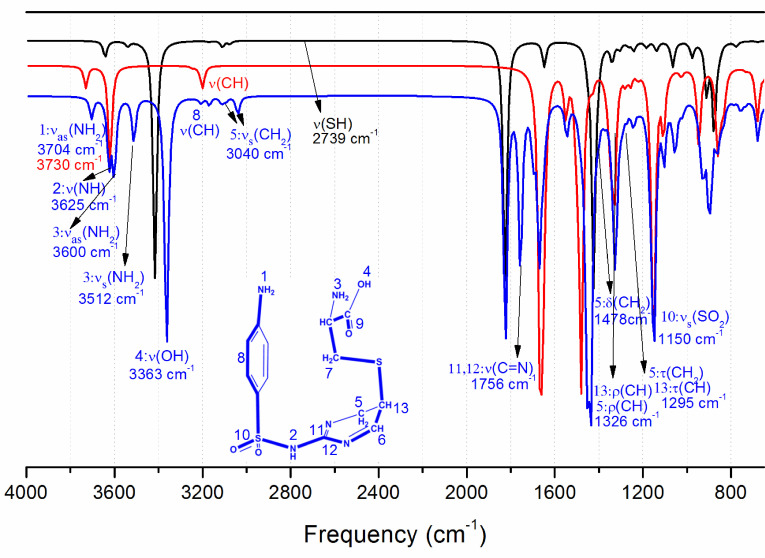
Calculated IR spectra of CYS, SD, and SD-CYS complex at ωB97XD/6-311++G(d,p) level.

Calculated IR spectra of the investigated molecules are displayed in Figure 7. Selected stretching vibrations are shown in the figure for the molecules. With addition of CYS to the pyrimidine part of the SD molecule, the S-H stretching vibration (2739 cm−1) of CYS disappeared and new vibrations appeared in the complex SD-CYS formation. Selected vibrational frequencies of the investigated molecules are given in Table S4 in detail. Some experimental vibrational bands are also included for comparison. New vibrations due to the distortions in pyrimidine at 3040 cm−1 and 3113 cm−1 appeared in the SD-CYS complex, corresponding to CH2 symmetrical (vs (CH2)) and asymmetrical (vas (CH2)) vibrations, respectively. These peaks agree quite well with the peak observed at 2819 cm−1 experimentally. Computed v (C=N) peaks at 1756 cm−1 and 1698 cm−1 also agree with experimentally observed peaks at 1621 cm−1 and 1585 cm−1 . Additionally, computed vibrational peaks in the same region of the molecule at 1478 cm−1 δ (CH2) and 1326 cm−1 ρ(C-H) are in agreement with experimentally observed peaks at 1383 cm−1 and 1298 cm−1 .

Observed peaks in the calculated and experimental IR spectra display shifts for the frequencies for the same vibrations as computational vibrational frequencies were not scaled. Another reason for the observed shifts may be that the experimental measurements were taken in the solid state, whereas computations were performed in solution. Although there are shifts in the IR peak values, the peaks with the same nature confirm that the formed complex has a S-bridged structure as experimentally predicted.

**Figure 8:**
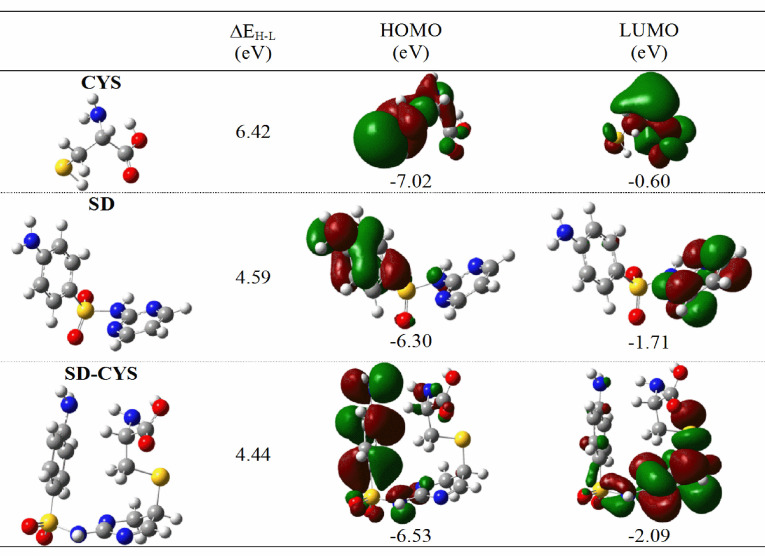
Frontier molecular orbitals, their energies, and HOMO-LUMO energy gaps for the compounds CYS, SD, and SD-CYS calculated at B3LYP/6-311++G(d,p) level in water.

We focus on the frontier HOMO and LUMO orbitals for determining chemical stability. Koopmans’ theorem [68] states that the ionization potential (IP) and electron affinity (EA) are related to the orbital energies of HOMO and LUMO: EA: –ELUMO ; IP: –EHOMO . Those molecular orbitals and orbital energy gaps of SD and SD-CYS were calculated at the B3LYP/6-311++G(d,p) level and are given in Figure 8.

The HOMO-LUMO gaps are larger in hard compounds and they are more stable and less reactive than in soft compounds with smaller HOMO-LUMO gaps. A small HOMO-LUMO gap allows transitions to excited states more easily; therefore, the electron density of soft molecules will change more easily compared to hard molecules. The conceptual DFT approach can provide information on molecular structure stability and reactivity [69].

Additionally, the absolute softness (σ) , chemical hardness (η) , and absolute electronegativity (χ) of the molecules were calculated at the same level and are listed in Table 2. The chemical hardness is a good indicator of chemical stability and can be used as a measure for the stability and reactivity of chemical compounds. As a rule of thumb, soft molecules are more polarizable than hard ones. The absolute electronegativity (χ) [70], chemical hardness (η) [71–73], and absolute softness were obtained by using the formulae χ = (IP + EA)/2, η = (IP − EA)/2, and s = 1/η , respectively. In addition, the electrophilicity index [74] (ω, global reactivity descriptor of molecules, as μ2 /2η , where μ is the chemical potential: μ = −(IP + EA)/2) [75] was calculated. In general, the electrophiles have a tendency to accept electrons and may form bonds with nucleophiles. Thus, electrophilicity is also a useful depicter for the analysis of chemical reactivity.

**Table 2:** 

	CYS	SD	SD-CYS
E_*HOMO*_(eV)	-7.02	-6.29	-6.53
E_*LUMO*_(eV)	-0.60	-1.71	-2.09
ΔE_*H-L*_ (eV)	6.42	4.58	4.44
IP (eV)	7.02	6.29	6.53
EA (eV)	0.60	1.71	2.09
χ (eV)	3.81	4.00	4.31
η (eV)	3.21	2.29	2.22
σ (eV^-1^)	0.31	0.44	0.45
μ (eV)	-3.81	-4.00	-4.31
ω (eV)	2.26	3.57	4.18

CYS and SD have higher stability and chemical hardness than SD-CYS under high excitation energies. The IP values of the SD-CYS molecule are not the lowest, but with the addition of cysteine to SD, the electron affinity of the SD-CYS system increases. The electrophilicity index of the complex is the highest.

**Figure 9:**
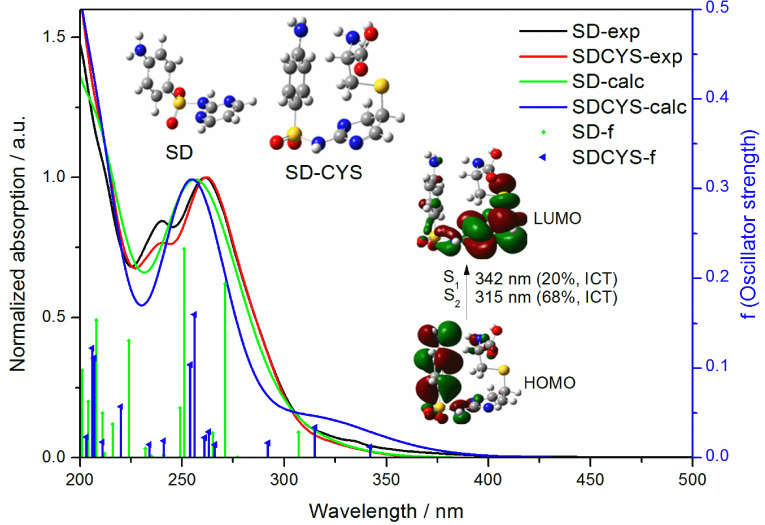
Experimental and calculated UV-Vis absorption spectra of SD and SD-CYS in water.

**Figure 10:**
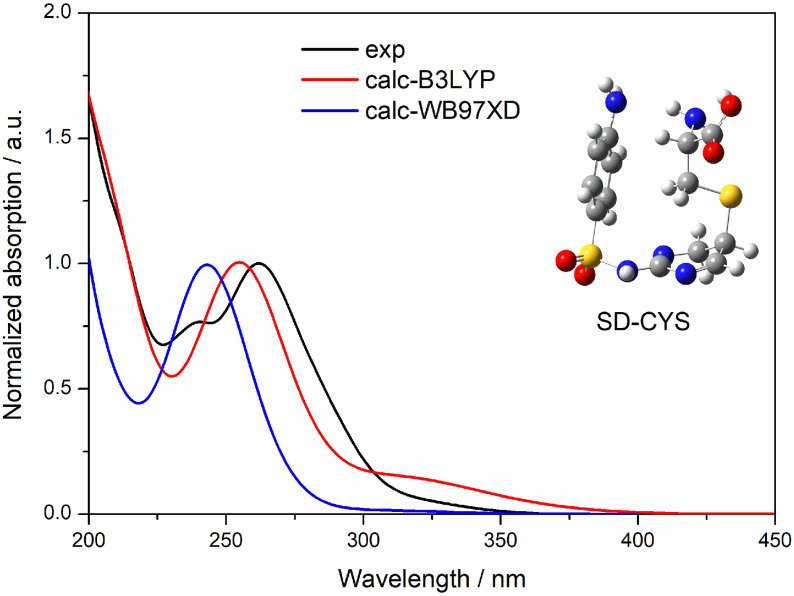
Comparison of experimental and calculated (with different functionals) UV-Vis absorption spectra of SDCYS in water.

UV-Vis absorption spectra of SD (5.2 ×10−5 M) and SD-CYS (1.9 ×10−3 M) in water were obtained experimentally and computationally with time-dependent density functional theory (TD-DFT) and the spectra are presented in Figure 9. Figure 10 shows the differences of UV-Vis absorption spectra between the calculated spectra with different functionals (B3LYP and ωB97XD) and the experimental one. B3LYP results are used in discussion as they agree better with the experimental spectra.

Calculated wavelengths in water are given in Table 3 for SD-CYS and Table S5 for SD in detail. Comparing the S0 →S1 wavelengths of SD and SD-CYS in water, a red shift of 35 nm was observed. The long wavelength absorption peak (342 nm) of the SD-CYS complex belongs to the transition between HOMO/HOMO-1 and LUMO orbitals, and it has an intramolecular charge transfer from aniline to pyrimidine and local excitation of pyrimidine characters. SD has S1 transition at 307 nm, which is assigned to the intramolecular charge transfer between aniline and pyrimidine parts (ICT1) between HOMO and LUMO (Figure S2).

**Table 3:** Excitation energies (ΔE), wavelengths (λex) , transition dipole moments (μtr) , oscillator strengths (f), excitation character, and involved transition molecular orbitals and their contributions for SD-CYS in water at B3LYP/6-311++G(d,p) level.

State	ΔE (eV)	λ_*ex*_ (nm)	μ_*tr*_ (D)	f	Character^a^	Predominant transitions	%
S_1_	3.62	342	0.1379	0.0122	LE1 ICT1	H-1→L H→L	64 20
S_2_	3.93	315	0.3510	0.0338	ICT1 LE1	H→L H-1→L	68 20
S_3_	4.25	292	0.1590	0.0165	LE1,ICT1 LE1	H-2→L H-4-L	54 38
S_4_	4.66	266	0.1275	0.0146	LE1 ICT1,LE1	H-4→L H-2→L	50 38
S_5_	4.72	263	0.2499	0.0289	LE(phenyl) ICT3	H→L+2 H-3→L	50 33
S_6_	4.76	261	0.1928	0.0225	ICT3 LE2	H-3→L H→L+1	60 20
S_7_	4.85	256	1.3452	0.1598	LE2 LE1	H→L+1 H-1→L+1	52 32
S_8_	4.89	254	0.8659	0.1037	LE1 LE2	H-1→L+1 H→L+1	59 34
S_10_	5.15	241	0.1499	0.0189	ICT2	H-1→L+2	69
S_26_	5.99	207	0.7585	0.1113	ICT4,LE1 ICT5,LE3	H-8→L H-5→L+1	48 29
S_28_	6.03	206	0.8272	0.1222	ICT5,LE3 ICT4,LE1	H-5→L+1 H-8→L	46 24
S_30_	6.10	203	0.1539	0.0230	ICT6	H-1→L+5	55

^a^ ICT1: Intramolecular charge transfer from aniline to pyrimidine part; LE1: local excitation of pyrimidine part; LE2: local excitation of aniline; ICT2: intramolecular charge transfer from pyrimidine part to aniline; ICT3: intramolecular charge transfer from CYS and phenyl to pyrimidine part; ICT4: intramolecular charge transfer from CYS and aniline to pyrimidine; ICT5: intramolecular charge transfer from CYS to aniline; ICT6: intramolecular charge transfer from pyrimidine part and S to CYS.

In contrast to SD, SD-CYS displayed ICT1 at 315 nm (S2 transition) between HOMO and LUMO orbitals, too. The absorption peaks observed at long wavelengths (342 nm, 315 nm) belong to the charge transfer from aniline to pyrimidine; unfortunately, these peaks do not appear in the experimental spectra as their oscillator strength values are too small. Experimental and calculated peaks at 260 nm can be local excitation of aniline (LE2). The significant peak of SD at 240 nm observed in the experimental UV spectrum was described as local excitation of pyrimidine (LE1) by computational results. CYS has its absorption band at wavelengths shorter than 250 nm; therefore, its effect at longer wavelengths is not significant (spectra are not shown). Due to the U shape of SD-CYS, CYS and the NH2 group at the opposite terminal are close to each other (distance between N in CYS and N in aniline = 3.22 Å). As a result, it has contributions to electronic transitions of SD-CYS at 207 and 206 nm (calculated) in the form of intramolecular charge transfer from cysteine to aniline part (ICT5). Additionally, there are other intramolecular charge transfers including cysteine: from CYS and phenyl to the pyrimidine part (ICT3, 261 nm), from CYS and aniline to the pyrimidine part (ICT4, 207 nm), and from the pyrimidine part and S to CYS (ICT6, 203 nm).

### 3.4. Conclusions

In this study, adduct formation between SD and CYS was confirmed by experimental and computational methods. Voltammetric measurements showed positive shifting at the peak potential of mercurous cysteine thiolate in the presence of SD, which revealed that a product formed from the fast follow-up reaction. Depending on the reactants and confirmed product, a reaction mechanism in which the CYS thiol group is added to the C(5) = C(6) double bond of the pyrimidine on SD by a nucleophilic attack is suggested.

DFT results have revealed that the S-bridged SD-CYS complex is more stable than the NH2 -bridged complex, as predicted by experimental results. Structural, electronic, and spectroscopic properties of the SDCYS complex were calculated by using DFT and TD-DFT methods and the results were in quite good agreement with the experimental results. The calculated electrophilicity index of the complex is the highest among all studied systems. The calculated ΔE and ΔΔG values indicate that the adduct formation reaction is endergonic and requires energy, in agreement with the experimental procedure. Computations also indicate that SD and its derivatives may effectively bind CYS and can be used to develop new molecules/drugs to target CYS.

Supplementary Materials
