# Supplementary material for: Combined computational and experimental studies on cysteine-sulfadiazine adduct formation
Source: Turk J Chem. 2020 Apr 1;44(2):502–17. doi: 10.3906/kim-1908-62 (PMC7671221; doi:10.3906/kim-1908-62)
Supplement: Supplementary file 1 — Supplementary Materials [file turkjchem-44-502-sup001.pdf]

Supporting Information

Table S1. Conformer analysis of SD-CYS (NH_2-bridged) in water at ωB97XD/6-311++G(d,p) level.

SD-CYS<br>(NH2-bridged)	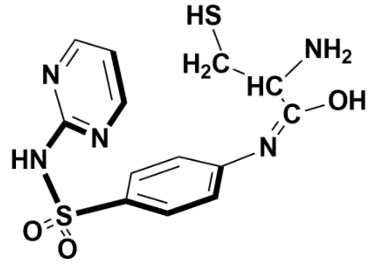	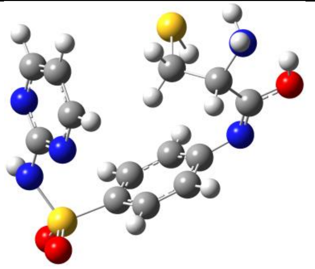<br>wSDCYS1C57	
Conformers	E+ZPE (a.u.)	Conformers	E+ZPE (a.u.)
wSDCYS1C01	–1799.857619	wSDCYS1C53	–1799.858785
wSDCYS1C02	–1799.857923	wSDCYS1C54	–1799.860172
wSDCYS1C03	–1799.857778	wSDCYS1C55	–1799.853048
wSDCYS1C04	–1799.856394	wSDCYS1C57*	–1799.862079
wSDCYS1C06	–1799.855928	wSDCYS1C58	–1799.853294
wSDCYS1C08	–1799.861613	wSDCYS1C60	–1799.854521
wSDCYS1C09	–1799.857808	wSDCYS1C62	–1799.854614
wSDCYS1C10	–1799.856410	wSDCYS1C64	–1799.850236
wSDCYS1C14	–1799.862024	wSDCYS1C65	–1799.850177
wSDCYS1C16	–1799.854602	wSDCYS1C66	–1799.855575
wSDCYS1C17	–1799.854966	wSDCYS1C68	–1799.854975
wSDCYS1C18	–1799.858853	wSDCYS1C71	–1799.853785
wSDCYS1C20	–1799.855639	wSDCYS1C72	–1799.855175
wSDCYS1C22	–1799.859618	wSDCYS1C73	–1799.850921
wSDCYS1C24	–1799.859775	wSDCYS1C74	–1799.853304
wSDCYS1C25	–1799.857434	wSDCYS1C75	–1799.851125
wSDCYS1C26	–1799.857400	wSDCYS1C76	–1799.858189
wSDCYS1C27	–1799.856527	wSDCYS1C77	–1799.855499
wSDCYS1C28	–1799.855612	wSDCYS1C78	–1799.860584
wSDCYS1C29	–1799.858384	wSDCYS1C80	–1799.858192
wSDCYS1C30	–1799.855519	wSDCYS1C82	–1799.850320
wSDCYS1C32	–1799.854021	wSDCYS1C83	–1799.851978
wSDCYS1C33	–1799.855113	wSDCYS1C84	–1799.851113
wSDCYS1C34	–1799.855670	wSDCYS1C85	–1799.851268
wSDCYS1C36	–1799.854129	wSDCYS1C86	–1799.855703
wSDCYS1C37	–1799.854129	wSDCYS1C88	–1799.855277
wSDCYS1C38	–1799.856958	wSDCYS1C90	–1799.857155
wSDCYS1C40	–1799.856226	wSDCYS1C91	–1799.856692
wSDCYS1C42	–1799.853976	wSDCYS1C92	–1799.851112
wSDCYS1C43	–1799.861612	wSDCYS1C93	–1799.854595
wSDCYS1C44	–1799.854291	wSDCYS1C94	–1799.854475
wSDCYS1C45	–1799.855978	wSDCYS1C95	–1799.855486
wSDCYS1C46	–1799.852675	wSDCYS1C96	–1799.855456
wSDCYS1C47	–1799.857649	wSDCYS1C97	–1799.855499
wSDCYS1C48	–1799.852740	wSDCYS1C98	–1799.860584
wSDCYS1C50	–1799.852836	wSDCYS1C99	–1799.851216
wSDCYS1C52	–1799.853001	wSDCYS1C100	–1799.853865

*H2O (E+ZPE): –76.4188; E+ZPE (SD-CYS (NH2-bridged))= –1876.2808 a.u.

Table S2. Conformer analysis of SD-CYS (SH-bridged) in gas phase and in water at ωB97XD/6-311++G(d,p) level.

SD-CYS	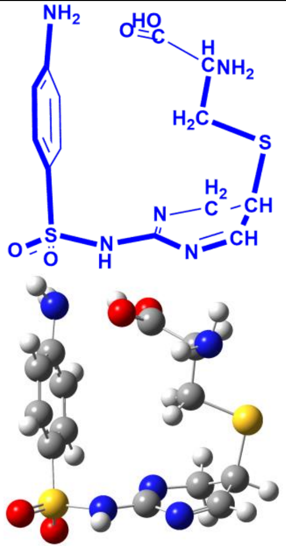 wSDCYS2C26	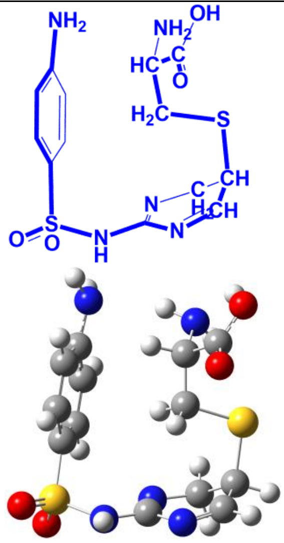 wSDCYS2C21
Conformers	E+ZPE (a.u.) (gas)	E+ZPE (a.u.) (water)
wSDCYS2C01	–1876.245250	–1876.281808
wSDCYS2C02	–1876.250939	–1876.284542
wSDCYS2C04	–1876.249825	–1876.282177
wSDCYS2C06	–1876.245081	–1876.281374
wSDCYS2C08	–1876.249201	–1876.281223
wSDCYS2C09	–1876.250385	–1876.280140
wSDCYS2C10	–1876.240627	–1876.279351
wSDCYS2C12	–1876.244753	–1876.278274
wSDCYS2C13	–1876.244614	–1876.278690
wSDCYS2C14	–1876.250943	–1876.284576
wSDCYS2C15	–1876.247978	–1876.283097
wSDCYS2C16	–1876.254737	–1876.287237
wSDCYS2C17	–1876.245108	–1876.280810
wSDCYS2C18	–1876.245033	–1876.281369
wSDCYS2C20	–1876.247611	–1876.278342
wSDCYS2C21	–1876.251370	–1876.287711
wSDCYS2C22	–1876.247428	–1876.278336
wSDCYS2C24	–1876.247802	–1876.281786
wSDCYS2C25	–1876.247802	–1876.281786
wSDCYS2C26	–1876.252282	–1876.283502
wSDCYS2C27	–1876.244929	–1876.278333
wSDCYS2C28	–1876.251861	–1876.283071
wSDCYS2C29	–1876.244858	–1876.278391
wSDCYS2C30	–1876.248975	–1876.277174
wSDCYS2C31	–1876.243325	–1876.281114
wSDCYS2C32	–1876.252270	–1876.281741
wSDCYS2C33	–1876.247329	–1876.277994
wSDCYS2C34	–1876.249820	–1876.280965
wSDCYS2C36	–1876.244971	–1876.278228
wSDCYS2C38	–1876.241995	–1876.279783
wSDCYS2C39	–1876.251080	–1876.282796
wSDCYS2C40	–1876.245949	–1876.279683
wSDCYS2C41	–1876.246214	–1876.278817

wSDCYS2C43	–1876.242300	–1876.276294
wSDCYS2C44	–1876.245873	–1876.279503
wSDCYS2C45	–1876.245217	–1876.275756
wSDCYS2C46	–1876.243729	–1876.279334
wSDCYS2C47	–1876.242291	–1876.275978
wSDCYS2C48	–1876.248068	–1876.281679
wSDCYS2C50	–1876.242279	–1876.276150
wSDCYS2C51	–1876.247367	–1876.278433
wSDCYS2C52	–1876.237486	–1876.273056
wSDCYS2C54	–1876.244759	–1876.276291
wSDCYS2C56	–1876.245878	–1876.278227
wSDCYS2C57	–1876.243229	–1876.278256
wSDCYS2C58	–1876.243668	–1876.276064
wSDCYS2C60	–1876.244689	–1876.276322
wSDCYS2C61	–1876.254601	–1876.281337
wSDCYS2C62	–1876.244645	–1876.279405
wSDCYS2C63	–1876.243364	–1876.280340
wSDCYS2C64	–1876.244483	–1876.279343
wSDCYS2C65	–1876.241987	–1876.277654
wSDCYS2C66	–1876.248145	–1876.280694
wSDCYS2C68	–1876.243870	–1876.277703
wSDCYS2C70	–1876.242973	–1876.276251
wSDCYS2C71	–1876.248077	–1876.281679
wSDCYS2C72	–1876.238488	–1876.273787
wSDCYS2C73	–1876.239125	–1876.274895
wSDCYS2C74	–1876.243611	–1876.279408
wSDCYS2C75	–1876.248083	–1876.282149
wSDCYS2C76	–1876.247909	–1876.276172
wSDCYS2C77	–1876.238480	–1876.273883
wSDCYS2C78	–1876.243164	–1876.275422
wSDCYS2C80	–1876.247349	–1876.276365
wSDCYS2C81	–1876.239416	–1876.278313
wSDCYS2C82	–1876.245977	–1876.279702
wSDCYS2C83	–1876.246466	–1876.280162
wSDCYS2C84	–1876.242857	–1876.275697
wSDCYS2C85	–1876.236861	–1876.279109
wSDCYS2C86	–1876.237456	–1876.279115
wSDCYS2C87	–1876.246329	–1876.277570
wSDCYS2C88	–1876.244460	–1876.277436
wSDCYS2C89	–1876.243652	–1876.277075
wSDCYS2C90	–1876.238715	–1876.276802
wSDCYS2C92	–1876.247477	–1876.279228
wSDCYS2C93	–1876.239962	–1876.273273
wSDCYS2C94	–1876.247904	–1876.280247
wSDCYS2C95	–1876.236688	–1876.279109
wSDCYS2C96	–1876.243396	–1876.278041
wSDCYS2C97	–1876.245574	–1876.274370
wSDCYS2C98	–1876.244372	–1876.276286
wSDCYS2C99	–1876.241391	–1876.272381
wSDCYS2C100	–1876.244291	–1876.277273

Table S3. Dipole moments (μ), sum of total electronic energies and zero point energies (E+ZPE), sum of electronic energies and free energies (E+ΔG, Hartree), complexation energy (ΔE), complexation free energy changes (ΔΔG), and selected geometrical parameters of investigated compounds calculated at ωB97XD/6-311++G(d,p) level in water

	CYS	SD	SD-CYS<br>S-bridge
μ (D)	6.22	11.2	16.7
E+ZPE (Hartree)	–721.8535	–1154.4459	–1876.2877
E+ΔG (Hartree)	–721.8864	–1154.4889	–1876.3401
[a]ΔE (kcal/mol)			7.34
[b]ΔΔG (kcal/mol)			22.09
Distances (Å)			
C2-S1		1.758	1.761
S1-N1		1.681	1.676
N1-C3		1.392	1.393
S2-C7	1.828	-	1.830
C7-C8	1.529	-	1.528
C5-S2	-	-	1.834
Angles (°)			
C2-S1-N1	-	106.64	106.43
S1-N1-C3	-	125.41	124.62
S2-C7-C8	114.12	-	109.75
Dihedral angles (φ°)			
C1-C2-S1-N1	-	74.74	96.84
C2-S1-N1-C3	-	49.88	–53.32
S1-N1-C3-N3	-	–159.64	–15.15
S1-N1-C3-N2	-	21.07	165.52
N1-C3-N3-C6	-	–178.73	179.89
N1-C3-N2-C4	-	179.48	–166.60
N2-C4-C5-S2	-	-	103.02
C4-C5-S2-C7	-	-	–64.06

[a]: ΔE = [E+ZPE(SD-CYS) – E+ZPE(SD) – E+ZPE(CYS)].
[b]: ΔΔG = [E+ΔG(SD-CYS) - E+ΔG(SD) - E+ΔG(CYS)].

Table S4. Selected vibrational frequencies (cm^{-1}) of SD-CYS, CYS, and SD at ground state by ωB97XD method with 6-311++G(d,p) basis set.

SD-CYS

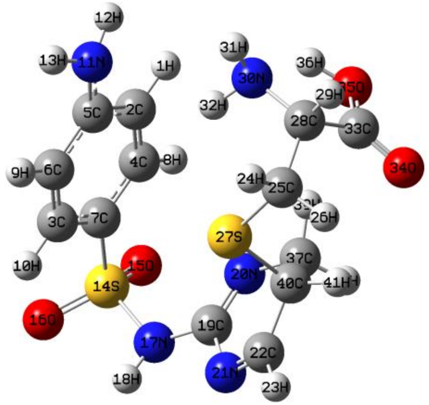

SD

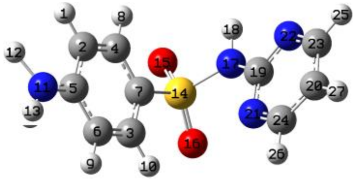

CYS

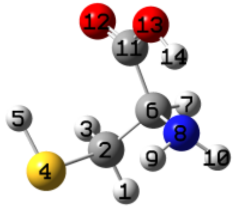

(cm^{-1})	SD-CYS		I	(cm^{-1})	SD		I	(cm^{-1})	CYS		I
3704 (3422)exp	1:ν_{as} (NH2) (aniline)	N(11)-H(12)-H(13)	62.01	3730	ν_{as} (NH2)	N(11)-H(12)-H(13)	62.96	3642	ν_{as} (NH2)	N(8)-H(9)-H(10)	45.30
3624	2:ν (N-H)	N(17)-H(18)	193.20	3619	ν (N-H)	N(17)-H(18)	186.23				
3600 (3353)exp	1:ν_s (NH2) 3:ν_{as} (NH2)	N(11)-H(12)-H(13) (an) N(30)-H(31)-H(32) (cys)	156.60	3616	ν_s (NH2)	N(11)-H(12)-H(13)	118.45	3540	ν_s (NH2)	N(8)-H(9)-H(10)	14.16
3512	3:ν_s (NH2)	N(30)-H(31)-H(32) (cys)	137.74					3416	ν (O-H)	N(13)-H(14)	704.75
								3171	ν_{as} (CH2)	C(2)-H(1)-H(3)	3.23
3363	4:ν (O-H)	N(35)-H(36)	827.36	3246	ν (C-H)	C(20)-H(27)	4.00	3110	ν_s (CH2)	C(2)-H(1)-H(3)	20.67
3203	8:ν (C-H)	C(2)-H(1) (an)	8.55					3076	ν (C-H)	C(6)-H(7)	10.11
3168	7:ν_{as} (CH2)	C(25)-H(24)-H(26) (cys)	7.02	3238	ν (C-H)	C(3)-H(10)	2.39	2740	ν (S-H)	S(4)-H(5)	0.52
3113	5:ν_{as} (CH2)	C(37)-H(38)-H(39) (pyr)	22.14	3215	ν (C-H) ν (C-H)	C(4)-H(8) C(2)-H(1)	6.01	1826	ν (C=O) ρ (O-H)	C(11)-O(12) O(13)-H(14)	1372.40
3040	5:ν_s (CH2)	C(37)-H(38)-H(39)	65.08	3202	ν (C-H)	C(24)-H(26)	26.67	1648	δ (NH2)	N(8)-H(9)-	154.13

(2819)exp		(pyr)								H(10)	
1824	9:ν (C=O)<br>4:ρ (O-H)	C(33)-O(34)<br>O(35)-H(36)	1467.82	3199	ν (C-H)	C(23)-H(25)	25.60	1450	δ (CH2)	C(2)-H(1)-H(3)	113.46
1756<br>(1621)exp	11:ν (C=N)<br>14:ν (C=N)<br>2:ρ (N-H)	N(20)-C(19) (pyr)<br>N(21)-C(22) (pyr)<br>N(17)-H(19)	1078.23	3198	ν (C-H)	C(6)-H(9)	14.66	1424	ρ (O-H)	O(13)-H(14)	1727.40
1698	14:ν (C=N)	N(21)-C(22) (pyr)	304.05	1668	δ (NH2)	N(11)-H(12)-H(13)	1569.83	1405	ρ (C-H)	C(6)-H(7)	101.78
1671	1:ν_s (CH2)	N(11)-H(12)-H(13) (an)	979.72	1651	ν (C-N)<br>ν (C=C)<br>ν (N-H)	C(19)-N(21)<br>C(24)-C(20)<br>N(17)-H(18)	607.31	1340	ω (CH2)	C(2)-H(1)-H(3)	143.16
1642	8:ν (C=C)	C(2)-C(5) (phen)<br>C(4)-C(7)	65.64	1639	ν (C=C)	C(5)-C(6)<br>C(4)-C(7)	78.74	1241	ν (C-O)	C(11)-O(13)	72.82
1546	ν (C-N)<br>8:ρ (C-H)<br>ρ (C-H)<br>ρ (C-H)<br>ρ (C-H)	C(5)-N(11)<br>C(6)-H(9)<br>C(3)-H(10)<br>C(2)-H(1)<br>C(4)-H(8)	241.24	1501	ρ (N-H)<br>ρ (C-H)<br>ρ (C-H)	N(17)-H(18)<br>C(20)-H(27)<br>C(24)-H(26)	404.04	1163	τ (NH2)	N(8)-H(9)-<br>H(10)	48.81
1488	7:δ (CH2)	C(25)-H(24)-H(26)	74.87	1486	ν (C=C)	C(3)-C(6)<br>C(2)-C(4)	161.90	1065	ρ (S-H)<br>ρ (C-H)	S(4)-H(5)<br>C(6)-H(7)	242.76
1478<br>(1383)exp	5:δ (CH2)	C(37)-H(38)-H(39)	27.23	1480	ν (C-N)<br>ρ (C-H)<br>ρ (N-H)	C(19)-N(17)<br>C(24)-H(26)<br>N(17)-H(18)	2163.37	976	ρ (S-H)	S(4)-H(5)	156.56
1454	2:ρ (N-H)	N(17)-H(18)	1711.92	1428	ρ (N-H)<br>ρ (C-H)	N(17)-H(18)<br>C(23)-C(20)	97.22	910	ρ (O-H)<br>ω (CH2)	O(13)-H(14)<br>N(8)-H(9)-<br>H(10)	566.24
1437	4:ρ (O-H) cys	O(35)-H(36)	2104.92	1360	ρ (N-H)<br>ρ (C-H)	C(24)-H(26)<br>N(17)-H(18)	79.29	877	ρ (O-H)<br>ω (CH2)	O(13)-H(14)<br>N(8)-H(9)-<br>H(10)	1082.98
1333	ν (C-N)<br>8:ρ (C-H)<br>8:ρ (C-H)	C(5)-N(11)<br>C(4)-H(8)<br>C(2)-H(1)	403.47	1348	ν (C-N)	C(5)-N(11)<br>C(4)-H(8)<br>C(2)-H(1)	484.65	854	ρ (S-H)<br>ν (C-O)	S(4)-H(5)<br>C(11)-O(13)	100.29
1326<br>(1298)exp	13:ρ (C-H)<br>5:ρ (C-H)	C(40)-H(41)<br>C(37)-H(38)	213.62	1329	ν_{as} (SO2)<br>ρ (C-H)	S(14)-O(15)-O(16)<br>C(23)-H(25)	1022.81				

1248	2:ρ (N-H)<br>13:ρ (C-H)	N(17)-H(18)<br>C(40)-H(41)	104.28	1256	ν (C=N)<br>ν (C=C)<br>ρ (N-H)	C(19)-N(22)<br>C(24)-C(20)<br>N(17)-H(18)	112.85				
1150<br>(1151)exp	10:ν_s (SO2)<br>2:ρ (N-H)	S(14)-O(15)-O(16)<br>N(17)-H(11)	2357.14	1152	ν_s (SO2)<br>ρ (C-H)	S(14)-O(15)-O(16)<br>C(4)-H(8)	2689.11				
				946	ν (S-N)	S(14)-N(17)	934.79				
				862	ω (C-H)<br>ω (C-H)<br>ω (C-H)<br>ω (C-H)<br>ν (S-N)	C(2)-H(1)<br>C(4)-H(8)<br>C(3)-H(10)<br>C(6)-H(9)<br>S(14)-N(17)	893.98				
				831	ω (C-H)<br>ω (C-H)<br>ω (C-H)	C(23)-H(25)<br>C(24)-H(26)<br>C(20)-H(27)	368.96				
				679	ν (S-C)	S(14)-C(7)	771.36				
				568	ω (SO2)	S(14)-O(15)-O(16)	2399				

ν: Stretching; δ: in-plane scissoring; ρ: in-plane rocking; τ: out-of-plane twisting; ω; out-of-plane wagging; s:symetrical; as: asymetrical.
Exp: Experimental; phen: pheyl; an: aniline; cys: cysteine; pyr: primidine; predicted vibrational frequencies were not scaled down with a factor.

Table S5. Vertical excitation energies (ΔE) corresponding to wavelengths (λ_{ex}), transition dipole moments (μ_{tr}), oscillator strengths (f), excitation character, and involved transition molecular orbitals and their percentage contributions for SD in water at B3LYP/6-311++G(d,p) level.

State	ΔE (eV)	λ_{ex} (nm)	μ_{tr} (D)	f	Character[a]	Predominant transitions	%
S_1	4.04	307	0.2874	0.0285	ICT1	H→L	70
S_2	4.47	277	0.0034	0.0004	LE1, ICT1<br>LE1, ICT1	H-2→L<br>H-3→L	58<br>33
S_3	4.58	271	1.7280	0.1940	LE2, ICT1	H→L+1	65
S_4	4.69	265	0.2399	0.0275	LE2	H→L+3	63
S_5	4.95	251	1.9220	0.2329	ICT1, LE1, LE2	H→L+2	63
S_6	4.97	249	0.4558	0.0556	LE1, ICT1	H-1→L	65
S_7	5.14	241	0.1218	0.0027	LE1, LE2<br>LE1, LE2	H-2→L+1<br>H-2→L+2	46<br>37
S_{10}	5.53	224	0.9632	0.1304	ICT2, LE1	H-1→L+1	65
S_{14}	5.87	211	0.3463	0.0498	LE1, LE2	H-3→L+1	58
S_{16}	5.96	208	1.0497	0.1532	ICT2, LE1	H-1→L+2	58
S_{18}	6.07	204	0.4223	0.0628	ICT2, LE2	H-2→L+3	62
S_{20}	6.17	201	0.6430	0.0972	LE1, LE2	H-2→L+2	57

[a] ICT1: Intramolecular charge transfer from aniline to pyrimidine; LE1: local excitation of pyrimidine; LE2: local excitation of aniline; ICT2: intramolecular charge transfer from pyrimidine to aniline.

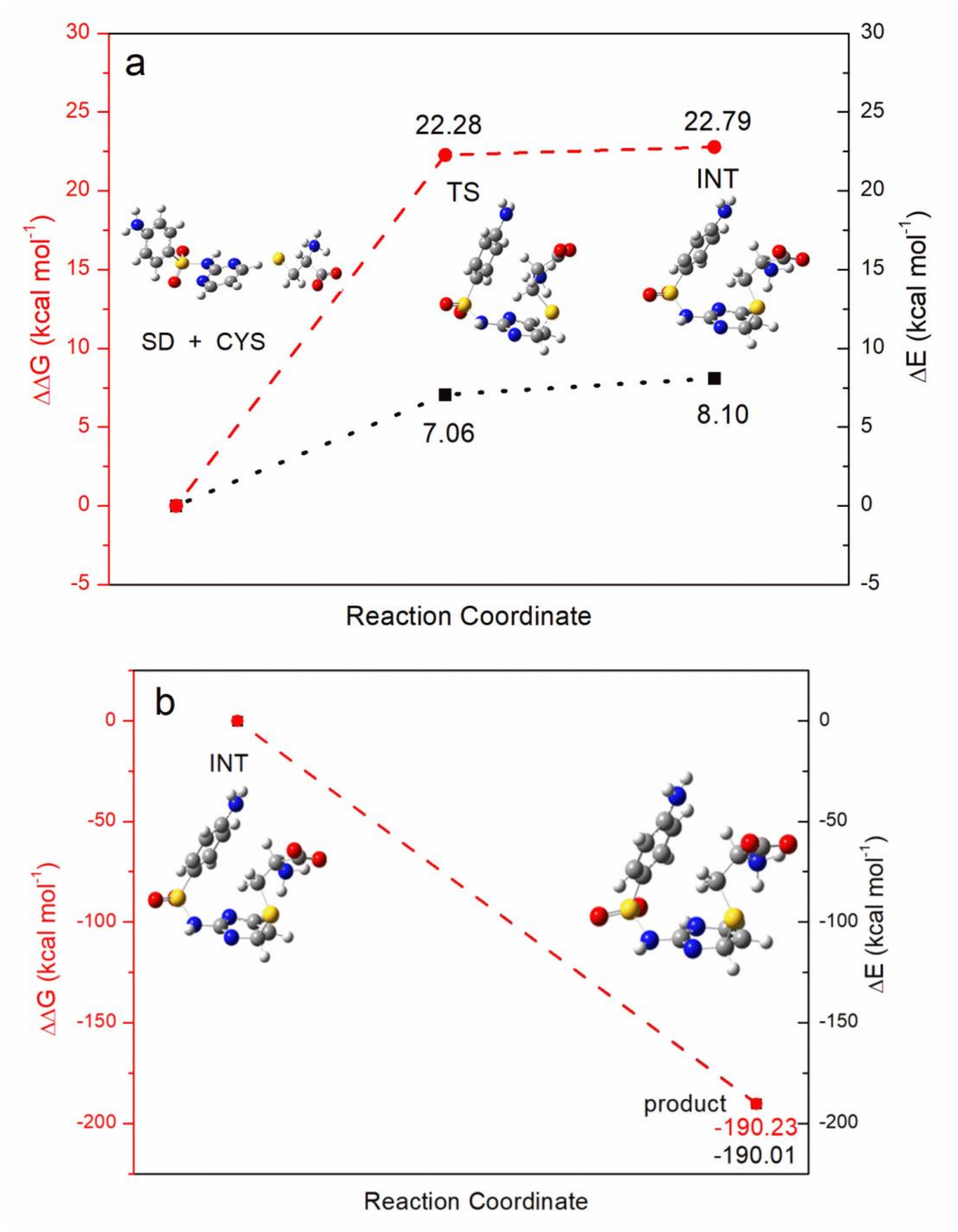

Figure S1. Calculated electronic (ΔE) and free energy (ΔΔG) differences for the steps: a) formation of INT from SD and CYS and b) formation of the product from INT.

	**SD**	**SD-CYS**
	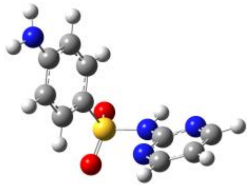	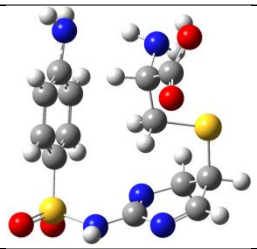
LUMO+5	-	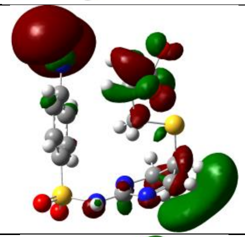
LUMO+3	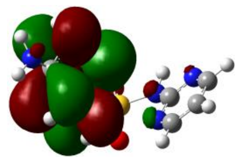	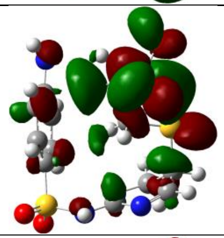
LUMO+2	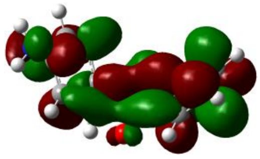	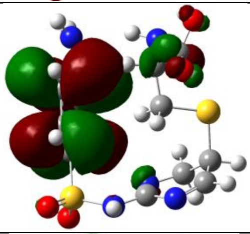
LUMO+1	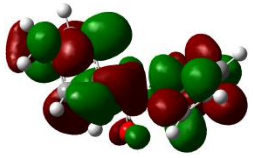	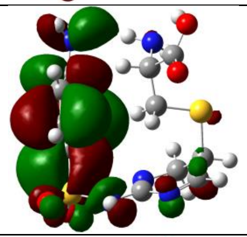
LUMO	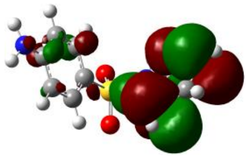	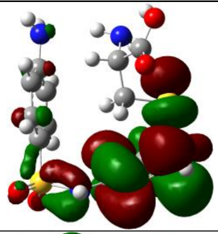
HOMO	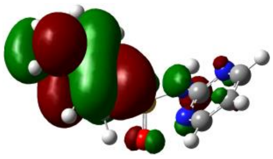	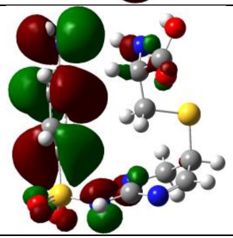

HOMO-1	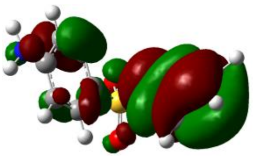	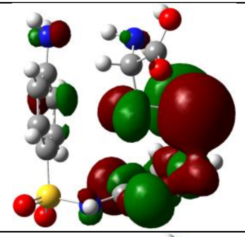
HOMO-2	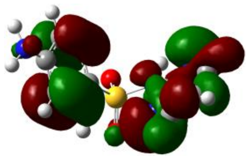	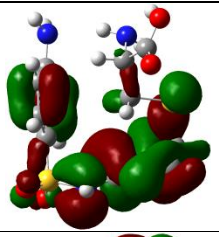
HOMO-3	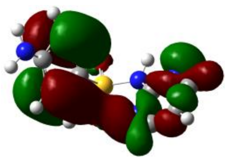	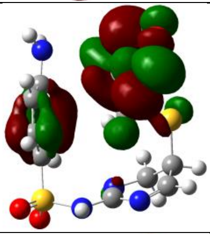
HOMO-4	-	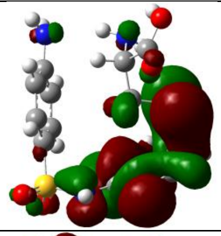
HOMO-8	-	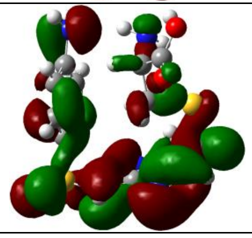

Figure S2. Selected molecular orbitals of SD and SD-CYS complex in water calculated with B3LYP/6-311++G(d,p).
